# Poster Session II – Poster of Distinction II - A214 ASSOCIATION BETWEEN CROHN’S DISEASE POLYGENIC RISK SCORE AND GUT MICROBIOME IN HEALTHY FIRST-DEGREE RELATIVES

**DOI:** 10.1093/jcag/gwaf042.214

**Published:** 2026-02-13

**Authors:** S Zezos, R Chen, M Bushra, Q Li, O Garcia, D Li, L Chen, H Q Huynh, R Panaccione, H Steinhart, K Jacobson, A Griffiths, W Turpin, K Croitoru, S Lee

**Affiliations:** IBD Center and Zane Cohen Centre for Digestive Diseases, Lunenfeld-Tanenbaum Research Institute, Toronto, ON, Canada; IBD Center and Zane Cohen Centre for Digestive Diseases, Lunenfeld-Tanenbaum Research Institute, Toronto, ON, Canada; Gastroenterology, University of Toronto, Toronto, ON, Canada; IBD Center and Zane Cohen Centre for Digestive Diseases, Lunenfeld-Tanenbaum Research Institute, Toronto, ON, Canada; Western University, London, ON, Canada; Cedars-Sinai Medical Center, Los Angeles, CA; Rutgers Robert Wood Johnson Medical School, New Brunswick, NJ; University of Alberta, Edmonton, AB, Canada; University of Calgary, Calgary, AB, Canada; IBD Center and Zane Cohen Centre for Digestive Diseases, Lunenfeld-Tanenbaum Research Institute, Toronto, ON, Canada; BC Children’s Hospital, Vancouver, BC, Canada; The Hospital for Sick Children, Toronto, ON, Canada; IBD Center and Zane Cohen Centre for Digestive Diseases, Lunenfeld-Tanenbaum Research Institute, Toronto, ON, Canada; IBD Center and Zane Cohen Centre for Digestive Diseases, Lunenfeld-Tanenbaum Research Institute, Toronto, ON, Canada; IBD Center and Zane Cohen Centre for Digestive Diseases, Lunenfeld-Tanenbaum Research Institute, Toronto, ON, Canada

## Abstract

**Background:**

Crohn’s Disease (CD) is characterized by a chronic, relapsing inflammation of the intestine of unknown cause. The current hypothesis is that microbial or environmental factors induce gut inflammation in genetically susceptible individuals, leading to chronic intestinal inflammation. The association between CD genetic risk and the gut microbiome as it relates to risk of CD development remains unclear.

**Aims:**

To investigate whether CD polygenic risk score (PRS) can predict CD onset and whether host genetic susceptibility to CD is associated with alterations in gut microbiome in first-degree relatives (FDRs).

**Methods:**

Stool samples and clinical metadata were collected from 2753 healthy FDRs of CD patients in the GEM cohort. Host genotype was measured using Immunochip, Exomechip and the Illumina Global Screening Array chip and imputed using the TOPMed Imputation Server. A CD-polygenic risk score (PRS) was derived based on previous IBD GWAS summary statistics. Subjects were stratified into quartiles (Q1-Q4) based on their PRS, with Q4 representing the highest PRS. 16S rRNA gene amplicon sequencing was performed to determine the relative abundance of genus-level taxa using the DADA2 pipeline. Fecal calprotectin (FCP) was measured by ELISA.

**Results:**

Among 2753 healthy FDRs, 78 developed CD during a median of 3.4 years from recruitment. Subjects in Q4 of the *CD-PRS* were more likely to develop CD than those in Q1: HR = 3.76 (95% CI: 1.73-8.17, p = 0.001). After adjusting for FCP, the increased risk remained significant for Q4 vs Q1 (HR = 2.23, 95% CI: 1.01-4.95, p = 0.048). No significant difference in Shannon alpha diversity was observed across PRS quartiles (p = 0.155). PERMANOVA indicated that PRS explained only 0.13% of beta diversity variation. CD PRS was associated with lower prevalence of *Izemoplasmatales*, and *Oscillospiraceae pseudoflavonifractor*, *Peptococcaceae uncultured*, *Lachnospiraceae roseburia*, *Ruminococcaceae*, *Oscillospiraceae UCG-005*, and *Oscillospiraceae UCG-003* and higher prevalence of *Carnobacteriaceae granulicatella* (q < 0.05). None of the taxa significantly modified or mediated the relationship between PRS and CD risk.

**Conclusions:**

CD-PRS is significantly associated with increased risk of CD among FDRs, but not with gut microbial alpha or beta diversity. While certain individual taxa are associated with higher PRS, there was no evidence of interaction or mediating effect of the gut microbiome with PRS on CD risk.

**Funding Agencies:**

CCC, CIHRHelmsley Charitable Trust

